# The Predictive Role of Social Intelligence Dimensions on Critical Thinking Dispositions Among Nurses: A Cross‐Sectional Study in Western Iran

**DOI:** 10.1002/nop2.70676

**Published:** 2026-07-24

**Authors:** Nahid Sourni, Mahvash Kahrizi, Nader salari, Maryam Janatolmakan, Rostam Jalali

**Affiliations:** ^1^ Department of Nursing, School of Nursing and Midwifery Kermanshah University of Medical Sciences Kermanshah Iran; ^2^ Islamic Education, Department of Islamic Studies School of Medicine Kermanshah University of Medical Sciences Kermanshah Iran; ^3^ Biostatistics, Health Policy and Promotion Institute Kermanshah University of Medical Sciences Kermanshah Iran; ^4^ Lifestyle Modification Research Center, Health Institute Kermanshah University of Medical Sciences Kermanshah Iran; ^5^ Social Development and Health Promotion Research Center, Health Institute Kermanshah University of Medical Sciences Kermanshah Iran

**Keywords:** critical thinking, Iran, nurses, professional competence, regression analysis, social intelligence

## Abstract

**Aims:**

This study aimed to determine the predictive role of specific social intelligence dimensions on nurses' critical thinking dispositions in western Iran.

**Design:**

A cross‐sectional descriptive correlational design.

**Methods:**

This study was conducted among 442 nurses from eight teaching hospitals affiliated with Kermanshah University of Medical Sciences, western Iran. Participants were selected via stratified random sampling. Data were collected using a demographic checklist, the 33‐item Ricketts Critical Thinking Disposition Assessment, and the 21‐item Tromsø Social Intelligence Scale. Data were analysed using descriptive statistics, independent t‐tests, one‐way ANOVA, Pearson correlation, multiple linear regression, and hierarchical regression analysis. Effect sizes (Cohen's *d*) were calculated where applicable. All statistical analyses were performed using SPSS version 26.

**Results:**

The findings revealed that overall critical thinking and social intelligence scores fell within the medium range; however, more than half of the participants demonstrated high‐level critical thinking, with innovativeness scoring the highest among its subscales. The overall correlation between the two constructs was weak and non‐significant, although intra‐dimensional relationships within each construct were strong and positive. Regression analyses indicated that innovativeness and cognitive maturity significantly predicted social information processing, while social skills and social awareness were positively predicted by innovativeness and cognitive maturity, respectively. Social information processing emerged as a negative predictor of critical thinking, whereas social awareness positively predicted cognitive maturity. Notably, overall social intelligence was not explained by any critical thinking dimensions, and in hierarchical analysis, only social information processing remained an independent predictor of critical thinking.

**Conclusion:**

The social intelligence, critical thinking relationship is dimension‐specific, not global. Total social intelligence did not predict critical thinking; however, social information processing negatively predicted it, whereas innovativeness and cognitive maturity positively influenced social dimensions. These findings challenge using global scores, suggesting leadership should prioritise cognitive competencies and educational interventions target specific social‐cognitive components.

**Implications for the Profession and/or Patient Care:**

Target cognitive competencies and specific social intelligence dimensions, not overall scores, to improve clinical reasoning and patient care.

**Public Contribution:**

Nurses from eight teaching hospitals affiliated with Kermanshah University of Medical Sciences voluntarily participated; no other public contribution was involved.

## 1 Introduction

As frontline clinicians, nurses require critical thinking to analyse complex situations, communicate effectively, and make safe, evidence‐informed decisions (Falcó‐Pegueroles et al. 2021; Lee and Chang 2022). Critical thinking is a reflective, logical cognitive process of analysis and evaluation, essential for clinical reasoning, problem‐solving and ethical decision‐making (Jintalan and Litao 2024; Farzi and Tarrahi 2023). Evidence indicates that higher critical thinking in nurses is associated with better clinical performance, outcomes and adaptability under pressure; consequently, this competency has been widely integrated into nursing curricula worldwide (Lee and Chang 2022; Farzi and Tarrahi 2023; Cárdenas‐Becerril et al. 2020).

However, cognitive skills alone are insufficient in the relational context of nursing. Social intelligence, the capacity to understand and navigate social interactions, encompassing empathy, emotional awareness, interpersonal sensitivity, and the ability to build therapeutic relationships has therefore gained growing recognition as a vital attribute for healthcare professionals (Lee et al. 2024; Savci et al. 2022). This is particularly evident in practice, as nurses with higher social intelligence demonstrate greater effectiveness in managing team dynamics, resolving conflicts and delivering patient‐centred care (Hassan Helaly et al. 2022; Ansari et al. 2022).

Emerging evidence suggests a complex, synergistic relationship between social intelligence and critical thinking, with network modelling identifying social skills as a pivotal integrating node (Al‐Adamat et al. 2026). However, this relationship is not unidirectional; improving critical thinking alone does not automatically enhance social intelligence, indicating they are distinct yet interacting constructs (Gorucu et al. 2025). Despite their recognised importance, existing literature has largely treated these constructs as monolithic total scores, overlooking how specific sub‐dimensions interact across diverse cultural and hierarchical contexts, a gap the present study aims to address.

In the Middle Eastern context, regional evidence remains fragmented and largely confined to nursing students, emphasising emotional intelligence over social intelligence and cognitive skills over thinking dispositions (Vahedi et al. 2015; Mousazadeh et al. 2021; Ayed et al. 2025). Although the Tromsø Social Intelligence Scale has been validated in Iran (Rezaie 2011), its specific sub‐dimensions remain unexplored among practicing nurses in relation to critical thinking. Consequently, how these sub‐dimensions interact under the unique pressures of the Iranian healthcare system is unclear. To address these gaps, the present study examined the association between social intelligence and critical thinking among hospital nurses in western Iran.

## 2 Purpose of the Study

Drawing on Bandura's Social Cognitive Theory (Bandura 1986), the Multidimensional Model of Social Intelligence (Silvera et al. 2001), and competency‐based nursing education (Welch and Smith 2022), this study examined how social intelligence dimensions (Sheldon and Ellington 2008) predict critical thinking dispositions among nurses and their variation across demographic/professional factors.

## 3 The Study Objectives

This study aimed to:

- Assess current levels of social intelligence and critical thinking.
- Examine bivariate and dimensional correlations between them.
- Identify which dimensions of social intelligence predict critical thinking and vice versa.
- Compare these competencies based on demographic and professional characteristics.

## 4 Method

### 4.1 Study Design and Settings

This cross‐sectional descriptive‐correlational study was conducted in 2024 among nurses working in hospitals affiliated with Kermanshah University of Medical Sciences, Iran. Data were collected from nurses employed in internal medicine, surgery, emergency, intensive care units (ICU/CCU), paediatrics, obstetrics and gynaecology, oncology, and education units across eight teaching hospitals in Kermanshah.

### 4.2 Participants and Sampling

#### 4.2.1 Eligibility Criteria and Sampling Method

The target population included all registered nurses with at least 1 year of clinical experience and a bachelor's degree or higher in nursing. Nurses who reported a diagnosed mental health disorder or were unwilling to participate were excluded.

A stratified random sampling method was employed to ensure proportional representation across the various teaching hospitals affiliated with Kermanshah University of Medical Sciences. The strata were defined based on hospital affiliation, with a total of eight hospitals included in the study. Within each hospital stratum, the sample size was determined proportionally to the total number of nursing staff employed at that site. For example, in one of the eight hospitals, which employed 697 nurses, 131 nurses (representing approximately 30% of the total sample) were allocated. Subsequently, nurses within each hospital stratum were randomly selected using simple random sampling from complete personnel lists. This process resulted in a total sample of 442 nurses selected from a total population of 2351 nurses across the eight participating hospitals. The sample size was calculated based on the formula for correlation studies, using a 95% confidence level (*α* = 0.05), 80% power, and a conservative expected correlation coefficient of *r* = 0.15 (informed by previous studies) (Ayed et al. 2025). The initial estimate was 402 participants, with an additional 10% added to account for potential non‐response or incomplete data, resulting in a final sample size of 442 nurses.

### 4.3 Instruments

The data collection tool consisted of three sections. The first section included a demographic checklist covering age, gender, marital status, educational level, work experience, shift pattern, department, employment status, average monthly working hours, and job rank. The second section was the Ricketts Critical Thinking Disposition Assessment (CTDA), developed by Ricketts to evaluate the level of inclination toward critical thinking (Ricketts and Rudd 2005).

This tool was specifically selected for its strong theoretical alignment with the study's focus, as it measures critical thinking as a tripartite disposition (cognitive engagement, cognitive maturity, and innovativeness) rather than merely logical reasoning, which is highly relevant to how nurses approach complex clinical environments. It includes 33 items evaluating the level of inclination toward CT, scored on a 5‐point Likert scale (1 = strongly disagree to 5 = strongly agree). To calculate the score of each subscale, the scores of all the items belonging to that subscale must be added and divided by the number of items. The Persian version of this inventory has been widely used and validated in recent Iranian studies. Mousazadeh et al. (2021) confirmed the psychometric properties of the scale in nursing students, reporting a Cronbach's alpha of 0.85 (Mousazadeh et al. 2021). Similarly, Barry et al. (2020) utilised this instrument in their study and reported a Cronbach's alpha of 0.80 for the CTDA (Barry et al. 2020). These findings confirm the acceptable internal consistency of the Persian version. In the present study, the Cronbach's alpha for the Ricketts questionnaire was calculated to be 0.85, demonstrating excellent reliability for this sample. The third section was the Tromsø Social Intelligence Scale (Silvera et al. 2001), a 21‐item instrument assessing social information processing, social skills, and social awareness. This scale was deliberately chosen over alternative instruments to ensure clear conceptual boundaries, as it theoretically distinguishes social intelligence, which focuses on the cognitive processing of external social information and behavioural skills in interactions, from emotional intelligence, which predominantly addresses intra‐personal emotional perception and regulation. The Persian version of the scale was validated by Rezaie (2011), with subscale Cronbach's alpha values ranging from 0.64 to 0.73. In a recent study among Iranian nurses, the total scale showed a Cronbach's alpha of 0.87, confirming its reliability in this context (Ansari et al. 2022).

### 4.4 Data Collection

After obtaining ethical approval and institutional permissions, the researcher visited each unit to introduce the study and invite eligible nurses. All participants provided written informed consent prior to participation. Data were collected from June to August 2024 using a self‐administered questionnaire completed in a private setting during nurses' shifts to ensure confidentiality and accuracy. Participation was voluntary, and participants were informed of their right to withdraw at any time without penalty. No incentives were provided.

### 4.5 Ethics Approval and Consent to Participate

This study was approved by the Ethics Committee of Kermanshah University of Medical Sciences (Code: IR.KUMS.REC.1403.026), and all procedures were conducted in accordance with the ethical standards of the institutional research committee and the 1964 Helsinki Declaration and its later amendments. Written informed consent was obtained from all participants prior to data collection, and participation was entirely voluntary. Confidentiality of data was rigorously maintained throughout the study, with no personal identifiers collected; participants were explicitly assured that their responses would remain anonymous and would not affect their employment status or professional evaluation.

### 4.6 Data Analysis

Data were analysed using IBM SPSS Statistics version 26. Descriptive statistics (frequencies, percentages, means, and standard deviations) summarised participant characteristics and main variables. Prior to parametric tests, normality was assessed using the Kolmogorov–Smirnov test, skewness, and kurtosis (all within ±2, *p* > 0.05), and homogeneity of variance was confirmed via Levene's test (*p* > 0.05). Pearson's correlation coefficient examined bivariate relationships. Independent *t*‐tests and one‐way ANOVA compared mean scores across demographic subgroups. Multiple linear regression was performed bidirectionally to predict critical thinking from social intelligence dimensions and vice versa. Additionally, hierarchical regression analysis determined the incremental predictive power of social intelligence dimensions on critical thinking. Multicollinearity was checked using Variance Inflation Factor (VIF < 5) and tolerance (> 0.2). Effect sizes (Cohen's *d*) were calculated where applicable. Statistical significance was set at *p* < 0.05 for all analyses.

### 4.7 Reporting Guideline

This study followed the STROBE reporting guideline for cross‐sectional studies. A completed checklist is provided as Supporting Information.

## 5 Findings

### 5.1 Demographic and Professional Characteristics of Participants

The study included 442 nurses with a mean age of 33.12 ± 5.17 years. The majority were female (57.5%), married (69.9%), and held a Bachelor of Science in Nursing (BScN) degree (89.1%). Most participants had non‐permanent employment status (79.9%) and worked in rotating shifts (77.6%). The majority were staff nurses (95.5%), employed in internal (31.0%) or surgical units (42.5%). The average monthly working hours were 113.07 ± 19.36 h (Table 1).

**TABLE 1:** Demographic and professional characteristics of participants (*N* = 442).

Variable		*n* (%)
Age (years), mean ± SD		33.12 ± 5.17
Gender	Female	254 (57.5)
	Male	188 (42.5)
Marital status	Married	309 (69.9)
	Single	133 (30.1)
Educational level	BScN	394 (89.1)
	MScN	48 (10.9)
Work experience (years), mean ± SD		9.4 ± 5.20
Employment status	Non‐permanent	353 (79.9)
	Permanent	89 (20.1)
Shift pattern	Rotating	343 (77.6)
	Fixed	99 (22.4)
Job rank	Staff nurse	422 (95.5)
	Head nurse/supervisor	20 (4.5)
Department	Internal medicine—surgery	140 (31.7)
	Emergency	104 (23.5)
	Intensive care unit (ICU/CCU)	92 (20.8)
	Paediatrics	11 (2.5)
	Women's health/obstetrics and gynaecology	48 (10.9)
	Education	25 (5.7)
	Oncology	22 (5.0)
Average monthly working hours (h), Mean ± SD		113.07 ± 19.36

*Note:* Data are presented as frequency (percentage) for categorical variables and mean ± standard deviation for continuous variables. Percentages may not sum to exactly 100% due to rounding.

Abbreviations: BScN, Bachelor of Science in Nursing; MScN, Master of Science in Nursing.

No statistically significant differences were observed in social intelligence or critical thinking scores based on age, gender, marital status, educational level, work experience, shift pattern, or unit of employment. However, staff nurses (mean = 100.01 ± 13.81) scored significantly higher in critical thinking than head nurses/supervisors (mean = 91.60 ± 13.69), *p* = 0.005 (Table 2). The effect size was moderate (Cohen's *d* = 0.62), indicating a practically meaningful difference between the two groups.

**TABLE 2:** Comparison of social intelligence and critical thinking scores across demographic and occupational characteristics of nurses (*N* = 442).

Variable		Social intelligence	*p*	Critical thinking	*p*
Age (years), mean ± SD		—	0.150	—	0.110
Gender	Female	84.79 ± 20.24	0.800	99.25 ± 14.21	0.370
	Male	84.25 ± 23.56		100.15 ± 13.49	
Marital status	Married	84.25 ± 21.85	0.350	97.32 ± 14.03	0.970
	Single	83.99 ± 21.77		97.36 ± 13.62	
Educational level	BScN	84.40 ± 21.36	0.740	99.56 ± 13.80	0.574
	MScN	85.48 ± 22.56		100.12 ± 14.83	
Work experience (years), mean ± SD		—	0.390	—	0.180
Employment status	Non‐permanent	84.92 ± 23.33	0.490	100.01 ± 13.67	0.228
	Permanent	83.10 ± 23.40		98.12 ± 14.77	
Shift pattern	Rotating	84.62 ± 21.40	0.620	99.69 ± 13.90	0.821
	Fixed	85.48 ± 23.30		99.45 ± 13.99	
Job rank	Staff nurse	84.64 ± 23.38	0.430	100.01 ± 13.81	0.005
	Head nurse/supervisor	80.70 ± 21.17		91.60 ± 13.69	
Department	Internal medicine—surgery	87.30 ± 20.69	0.210	98.62 ± 13.92	0.990
	Emergency	85.26 ± 21.61		97.23 ± 12.50	
	Intensive care unit (ICU/CCU)	80.92 ± 18.18		100.47 ± 14.09	
	Paediatrics	85.00 ± 24.17		100.72 ± 17.97	
	Women's health/obstetrics and gynaecology	84.93 ± 18.72		99.79 ± 14.68	
	Education	81.72 ± 20.17		97.56 ± 15.31	
	Oncology	78.90 ± 21.26		97.63 ± 14.79	
Average monthly working hours (h), Mean ± SD		—	0.260	—	0.150

*Note:* Data are presented as mean ± standard deviation. *p*‐values were calculated using independent *t*‐tests for binary variables and one‐way ANOVA for categorical variables with > 2 levels. Statistical significance was set at *p* < 0.05; no significant differences were found in social intelligence or critical thinking by hospital name across the eight hospitals.

### 5.2 Critical Thinking and Social Intelligence Scores

The total critical thinking score ranged from 33 to 165, with a mean of 97.88 ± 22.21 (59.3% of the maximum possible score). Although the overall mean score fell within the medium range, participants were distributed across three levels: low (33–66, 9.0%), medium (66–99, 43.2%), and high (99–165, 47.7%). This indicates that a substantial proportion of participants (47.7%) demonstrated high‐level critical thinking. Among the subscales, innovativeness scored the highest (37.71 ± 9.68), followed by cognitive Maturity (33.14 ± 7.13) and cognitive engagement (27.03 ± 7.26).

For social intelligence, the overall mean was 84.54 ± 21.80, with a possible range of 21 to 147. The subscale scores were as follows: social information processing (range: 7–49; 28.74 ± 8.27), social skills (range: 7–49; 28.19 ± 7.78), and social awareness (range: 7–49; 27.61 ± 7.71). A weak positive correlation was observed between total critical thinking and total social intelligence scores (*r* = 0.077, *p* > 0.05), which was not statistically significant (Table 3).

**TABLE 3:** Descriptive statistics for critical thinking and social intelligence dimensions, and correlation between total scores among nurses (*N* = 442).

Variable	Dimension/level	Mean ± SD	*n* (%)	Total score (mean ± SD)
Critical thinking	Innovativeness (11–55)	37.71 ± 9.68	—	97.88 ± 22.21 (33–165)
	Cognitive maturity (9–45)	33.14 ± 7.13	—	
	Cognitive engagement (13–65)	27.03 ± 7.26	—	
	Low (33–66)	—	40 (9.0)	
	Medium (66–99)	—	191 (43.2)	
	High (99–165)	—	211 (47.7)	
Social intelligence	Social information processing (7–49)	28.74 ± 8.27	—	84.54 ± 21.80 (21–147)
	Social skills (7–49)	28.19 ± 7.78	—	
	Social awareness (7–49)	27.61 ± 7.71	—	

*Note:* Pearson's correlation coefficient (*r* = 0.077, *p* > 0.05) indicates a weak, statistically non‐significant relationship between total scores of critical thinking and social intelligence.

### 5.3 Correlations Between Social Intelligence and Critical Thinking Dimensions

Within the social intelligence (SI) dimensions, social skills showed a strong positive correlation with social awareness (*r* = 0.68). The total score for this domain (SI Total) also demonstrated strong positive correlations with social skills (*r* = 0.50) and social awareness (*r* = 0.64). In contrast, social information processing exhibited a different pattern, showing a very strong negative correlation with social skills (*r* = −0.73) and a moderate negative correlation with social awareness (*r* = −0.42).

Within the cognitive thinking (CT) dimensions, all variables showed strong positive intercorrelations. Innovativeness was strongly and positively associated with cognitive maturity (*r* = 0.62) and cognitive engagement (*r* = 0.51). The CT Total score demonstrated very strong positive correlations with innovativeness (*r* = 0.90), cognitive maturity (*r* = 0.77), and cognitive engagement (*r* = 0.77), confirming high internal convergence among these dimensions.

**FIGURE 1:**
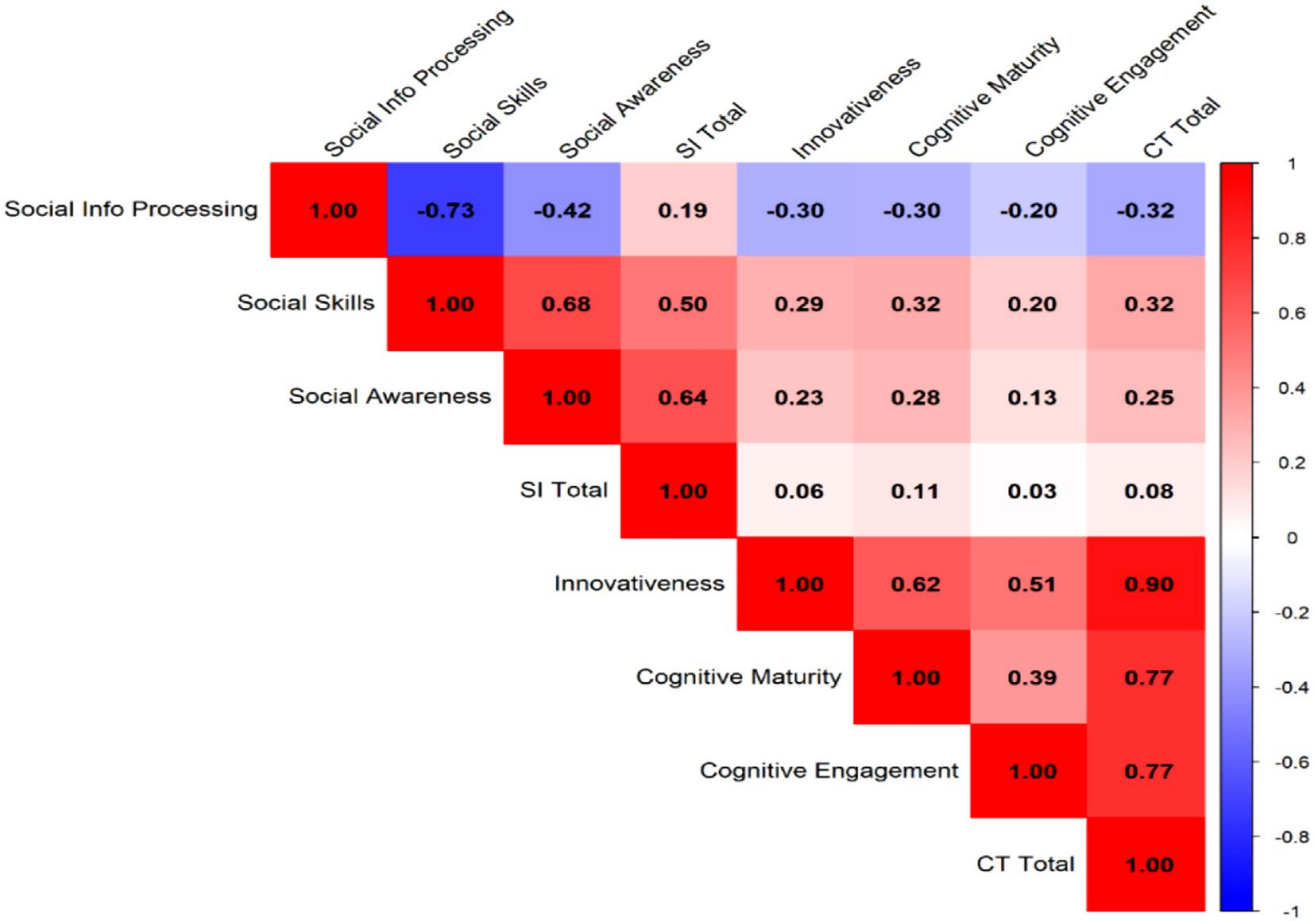
Heatmap representing the Pearson correlation matrix between social intelligence and cognitive thinking subscales.

In the between‐group relationships, social information processing displayed weak to moderate negative correlations with all cognitive thinking dimensions (ranging from −0.20 to −0.32). In contrast, social skills and social awareness showed weak to moderate positive correlations with cognitive thinking dimensions. The lowest between‐group association was observed between SI Total and cognitive thinking, suggesting that these two constructs operate largely independently of each other (Figure 1).

### 5.4 Multiple Regression Results Predicting Social Intelligence and Critical Thinking Dimensions

In the regression analysis predicting SI dimensions from CT dimensions, innovativeness and cognitive maturity both negatively and significantly predicted social information processing (*β* = −0.172, *p* = 0.005; and *β* = −0.172, *p* = 0.003, respectively). Social skills were positively predicted by innovativeness (*β* = 0.144, *p* = 0.020) and cognitive maturity (*β* = 0.210, *p* < 0.001). Social awareness was positively predicted only by cognitive maturity (*β* = 0.224, *p* < 0.001). Cognitive engagement was not a significant predictor in any model. *R* ^2^ values ranged from 0.082 to 0.117.

In the regression analysis predicting CT dimensions from SI dimensions, social information processing negatively and significantly predicted total CT (*β* = −0.203, *p* = 0.002), innovativeness (*β* = −0.201, *p* = 0.003), and cognitive maturity (*β* = −0.168, *p* = 0.012). Social awareness positively predicted cognitive maturity (*β* = 0.144, *p* = 0.020). Social skills were not significant predictors in any model. *R* ^2^ values ranged from 0.046 to 0.126. Notably, when the total social intelligence score was set as the dependent variable, none of the critical thinking dimensions emerged as significant predictors. This model was not statistically significant (*F* = 1.781, *p* = 0.150) with an *R* ^2^ of 0.005, indicating that the overall social intelligence construct is not explained by critical thinking dimensions (Table 4).

**TABLE 4:** Standardised regression coefficients (*β*) from multiple linear regression analyses predicting social intelligence dimensions from critical thinking dimensions and vice versa in nurses.

Dependent variable	Predictors	*B*	SE	*β*	*t*	*p*	*R* ^2^	Adjusted *R* ^2^	*F* (df)	*p* (*F*)
Social information processing	Constant	45.755	3.041		15.044	< 0.001	0.113	0.107	18.538 (3438)	< 0.001
	Innovativeness	−0.199	0.071	−0.172**	−2.796	0.005**				
	Cognitive maturity	−0.337	0.112	−0.172**	−3.001	0.003**				
	Cognitive engagement	−0.071	0.080	−0.047	−0.887	0.376				
Social skills	Constant	10.315	2.857		3.611	< 0.001	0.117	0.110	19.260 (3438)	< 0.001
	Innovativeness	0.156	0.067	0.144*	2.335	0.020*				
	Cognitive maturity	0.387	0.106	0.210**	3.663	< 0.001**				
	Cognitive engagement	0.060	0.076	0.042	0.796	0.427				
Social awareness	Constant	26.925	1.085		24.821	< 0.001	0.082	0.076	13.067 (3438)	< 0.001
	Innovativeness	0.037	0.025	0.091	1.459	0.145				
	Cognitive maturity	0.154	0.040	0.224**	3.833	< 0.001**				
	Cognitive engagement	−0.003	0.029	−0.006	−0.115	0.909				
Total social intelligence	Constant	83.090	2.863		29.018	< 0.001	0.005	−0.001	1.781 (3437)	0.150
	Innovativeness	−0.001	0.067	−0.001	−0.015	0.988				
	Cognitive maturity	−0.019	0.105	−0.010	−0.185	0.854				
	Cognitive engagement	0.012	0.075	0.008	0.155	0.877				
Critical thinking (total)	Constant	89.678	8.769		10.226	< 0.001	0.126	0.120	20.985 (3438)	< 0.001
	Social information processing	−0.342	0.111	−0.203**	−3.082	0.002**				
	Social skills	0.208	0.146	0.117	1.428	0.154				
	Social awareness	0.417	0.295	0.087	1.414	0.158				
Innovativeness	Constant	30.571	4.566		6.695	< 0.001	0.106	0.100	17.372 (3438)	< 0.001
	Social information processing	−0.174	0.058	−0.201**	−3.007	0.003**				
	Social skills	0.087	0.076	0.095	1.148	0.252				
	Social awareness	0.194	0.154	0.079	1.262	0.208				
Cognitive maturity	Constant	20.411	2.676		7.628	< 0.001	0.120	0.114	19.834 (3438)	< 0.001
	Social information processing	−0.086	0.034	−0.168*	−2.535	0.012*				
	Social skills	0.051	0.045	0.095	1.155	0.249				
	Social awareness	0.210	0.090	0.144*	2.330	0.020*				
Cognitive engagement	Constant	38.696	3.572		10.834	< 0.001	0.046	0.039	7.043 (3438)	< 0.001
	Social information processing	−0.082	0.045	−0.126	−1.823	0.069				
	Social skills	0.070	0.059	0.100	1.174	0.241				
	Social awareness	0.014	0.120	0.007	0.115	0.909				

*Note:* *N* = 442 for all analyses except those involving total social intelligence (*N* = 441). Predictors for the first four models: innovativeness, cognitive maturity, cognitive engagement. Predictors for the last four models: social information processing, social skills, social awareness. All variance inflation factors (VIF) were below 5, indicating no multicollinearity. *p < 0.05 (two‐tailed), **p < 0.01 (two‐tailed).

*p < 0.05 (two‐tailed).

**p < 0.01 (two‐tailed).

### 5.5 Hierarchical Regression Analysis Predicting Critical Thinking Disposition

In the hierarchical regression analysis predicting critical thinking disposition, in the first step (Model 1), Social information processing was entered as the initial predictor. This baseline model explained 6.8% of the total variance in critical thinking disposition (*R* ^2^ = 0.068, adjusted *R* ^2^ = 0.066) and yielded a statistically significant overall effect (*F*[1, 440] = 32.110, *p* < 0.001). At this step, social information processing showed a strong, significant inverse relationship with the dependent variable (*B* = −0.016, *β* = −0.261, *t* = −5.667, *p* < 0.001).

In the second step (Model 2), the remaining dimensions, namely social skills and social awareness, were introduced into the regression equation to isolate their incremental predictive power. Although the cumulative variance explained by the full model increased to 7.8% (*R* ^2^ = 0.078, adjusted *R* ^2^ = 0.072; *F*[3, 438] = 12.396, *p* < 0.001), the *R* ^2^ change indices indicated that adding these two dimensions added only 1.0% to the model's explanatory power, which was not statistically significant (Δ*R* ^2^ = 0.010, *F*_change[2, 438] = 2.435, *p* = 0.089).

In the final adjusted model (Model 2), Social information processing retained its position as the only statistically significant independent predictor (*B* = −0.010, *β* = −0.164, *t* = −2.413, *p* = 0.016). In contrast, after removing shared variance, the unique contributions of social skills (*β* = 0.112, *t* = 1.334, *p* = 0.183) and social awareness (*β* = 0.038, *t* = 0.598, *p* = 0.550) were not significant. Collinearity diagnostics for the final model showed variance inflation factor (VIF) values ranging from 1.899 to 3.346, which are well below the conservative threshold of 5.0, confirming that parameter estimates were not destabilised by multicollinearity (Table 5).

**TABLE 5:** Hierarchical multiple linear regression predicting critical thinking disposition from social intelligence dimensions (*N* = 442).

Predictors	*B*	SE	*β*	*t*	*p*	*R* ^2^	Adjusted *R* ^2^	*F* (df)	*p* (*F*)
Model 1 (Step 1)	—	—	—	—	—	0.068	0.066	32.110 (1, 440)	< 0.001
Constant	2.957	0.079		37.333	< 0.001				
Social information processing	−0.016	0.003	−0.261	−5.667	< 0.001				
Model 2 (Step 2)	—	—	—	—	—	0.078	0.072	12.396 (3, 438)	< 0.001
Constant	2.385	0.324		7.369	< 0.001				
Social information processing	−0.010	0.004	−0.164	−2.413	0.016				
Social skills	0.007	0.005	0.112	1.334	0.183				
Social awareness	0.007	0.011	0.038	0.598	0.550				

*Note:* *N* = Model 2 Change Statistics: Δ*R* ^2^ = 0.010, *F*_change(2, 438) = 2.435, *p* = 0.089. All VIFs < 5 (ranging from 1.899 to 3.346), indicating no severe multicollinearity.

## 6 Discussion

This cross‐sectional study examined the predictive role of social intelligence dimensions on critical thinking dispositions among nurses in western Iran. Regarding demographic and professional characteristics, critical thinking and social intelligence were largely unaffected by age, gender, education, or work experience, with one notable exception: staff nurses exhibited significantly higher critical thinking than head nurses and supervisors. This relative absence of demographic associations raises the possibility that clinical experience alone does not necessarily enhance these competencies, contrasting with Mousazadeh et al. (2021), who reported a positive correlation between critical thinking and age among nursing students (Mousazadeh et al. 2021). Such a discrepancy may suggest that while critical thinking develops during education, this trajectory may plateau in the professional workforce. Similarly, Barry et al. (2020) observed an inverse correlation between professional self‐concept and critical thinking, implying that workplace routines could neutralise age‐ or experience‐related advantages (Barry et al. 2020). Moreover, Gorucu et al. (2025) noted that clinical stress and structural factors may blunt socio‐cognitive development over time, offering a potential explanation for why tenure did not predict higher scores in our sample (Gorucu et al. 2025). In parallel, the finding that staff nurses outperformed leaders points to a possible ‘leadership paradox’, wherein administrative distance from bedside practice may attenuate cognitive dispositions. This aligns with Aqtam et al. (2026), who demonstrated that formal authority does not necessarily equate to superior cognitive competencies in Palestinian hospitals (Aqtam et al. 2026), and extends Hassan Helaly et al. (2022), who emphasised the influence of head nurses' social intelligence on staff outcomes (Hassan Helaly et al. 2022). Taken together, our data suggest that leaders' own cognitive competencies may be compromised, possibly because leadership advancement in Iran prioritises seniority over demonstrated capabilities.

A further finding was that critical thinking scores fell within the medium range, with nearly half of participants demonstrating high‐level critical thinking, appearing to exceed typical student samples in Iran (Bakhtiari‐Dovvombaygi et al. 2024). Innovativeness scored highest among subscales, followed by cognitive maturity and engagement. Social intelligence also showed moderate levels with balanced subscale distributions. These findings align with reports among nursing students (Gorucu et al. 2025), yet extend them by documenting that this moderate profile persists into professional practice. Notably, the prominence of innovativeness contrasts with Gorucu et al. (2025) who found systematicity and metacognition improved more following education (Gorucu et al. 2025), suggesting clinical workplaces may reward innovative problem‐solving over metacognitive awareness, a distinction not previously reported in Iran. Moreover, lower cognitive engagement relative to innovativeness points to a performance paradox: nurses appear innovative yet lack sustained cognitive involvement, an overlooked gap. The balanced social intelligence profile partially supports Kaya et al. (2017), who found emotional intelligence correlated with critical thinking through self‐motivation (Kaya et al. 2017). Although that study examined emotional intelligence, its finding that specific subcomponents, not global scores, drive the relationship appears consistent with our observation that social information processing, social skills and social awareness each contribute differently. Overall, these findings suggest that while Iranian nurses show capacity for higher‐order thinking, the innovativeness‐engagement gap may signal a need for workplace interventions targeting sustained cognitive involvement, a gap prior research has not explicitly addressed.

The strong inverse relationship between social information processing and social skills is among the more novel observations of this study, though it requires cautious interpretation. One possibility is that extensive analytical decoding of social cues may lead to cognitive overload, potentially suppressing more intuitive interpersonal responses (Heck et al. 2024; Elimari and Lafargue 2024). However, an alternative explanation is that the Social Information Processing subscale may partly reflect rumination or social anxiety rather than pure analytical competence, a distinction not fully resolved in previous factor analyses (Lee et al. 2024). Additionally, the weak overall correlation between total social intelligence and total critical thinking diverges from some student‐based studies (Abou Hashish and Bajbeir 2018; Aslani et al. 2025) but aligns with a recent trial reporting no improvement in social intelligence following critical thinking training (Gorucu et al. 2025). This discrepancy could reflect a developmental shift, wherein the relationship between these abilities weakens in demanding clinical environments where emotional and cognitive demands compete for attentional resources. Importantly, the weak global correlation does not negate meaningful dimension‐level associations, as our subcomponent analysis demonstrates (Al‐Adamat et al. 2026). Nevertheless, the strong negative finding between social information processing and social skills should be interpreted with caution, given potential common method bias (both constructs were self‐reported) and the cross‐sectional design, which precludes causal inference. A practical implication for nursing education may be the inclusion of dual‐processing training, exercises that alternate between analytical case analysis and time‐pressured simulation drills that encourage intuitive responding. Future longitudinal research is needed to examine whether these patterns persist over time.

Regarding predictive relationships, innovativeness and cognitive maturity showed opposing effects on social intelligence subcomponents: both negatively predicted social information processing while positively predicting social skills and awareness, a pattern that may explain why total social intelligence was not predictable from any critical thinking dimension, as these opposing effects may cancel each other out when aggregated. Social information processing consistently and negatively predicted multiple critical thinking dimensions, whereas social skills showed no predictive power in any model. These findings diverge from student‐based regression studies that reported uniformly positive effects (Roh 2024; Heo 2015) and contrast with models where emotional intelligence significantly predicted critical thinking (Vahedi et al. 2015; Omidi et al. 2019). Notably, cognitive engagement did not emerge as a significant predictor in any regression model. The weak global prediction, despite significant subcomponent‐level associations, suggests that reliance on total scores may obscure dimension‐specific relationships. Furthermore, hierarchical regression indicated that only social information processing remained an independent predictor of critical thinking, with social skills and social awareness adding no significant incremental explanatory power. The consistently modest explanatory power across models implies that the model fit is limited, suggesting that these constructs, while statistically significant at the dimensional level, may not be strong determinants in isolation. Taken together, these findings suggest that the relationship between social intelligence and critical thinking in practicing nurses is dimension‐specific, directionally heterogeneous, and appears to be context‐dependent, rather than a simple, uniformly positive association.

## 7 Conclusion

This study revealed that nurses' critical thinking scores fell within the medium range, with nearly half demonstrating high‐level critical thinking. Among demographic variables, job rank was the sole significant factor, with staff nurses outperforming head nurses and supervisors. No significant overall correlation was found between social intelligence and critical thinking; however, meaningful dimension‐specific associations emerged, with opposing predictive effects observed across dimensions. These findings suggest that the relationship between the two constructs is not global but dimension‐dependent. Accordingly, nursing curricula may benefit from targeted training that addresses specific cognitive and social dimensions rather than relying on general competency measures. Furthermore, leadership promotion criteria could be revised to incorporate cognitive competencies alongside seniority. Future research is needed to examine the causal mechanisms underlying these dimension‐specific associations.

## 8 Limitation

This study has several limitations that should be acknowledged. First, the cross‐sectional design precludes causal inference regarding the relationship between social intelligence and critical thinking. To establish causality, future research should employ longitudinal designs tracking how these competencies co‐evolve over time or quasi‐experimental studies evaluating the efficacy of specific educational interventions aimed at enhancing social intelligence and its subsequent impact on critical thinking. Second, regarding sample representativeness, data were collected via convenience sampling from public university hospitals in a single province (Kermanshah). Consequently, findings may not generalise to nurses in private hospitals, non‐academic healthcare settings, or other regions of Iran with different organisational cultures. Future studies should utilise multi‐centre, stratified random sampling to ensure broader representativeness. Third, data were self‐reported, which introduces the risk of social desirability bias, potentially inflating social intelligence scores. To mitigate this, future research should incorporate objective, observational assessments of critical thinking in clinical simulations or utilise 360‐degree feedback for social intelligence. Fourth, this study measured critical thinking disposition (the inclination or willingness to think critically) rather than critical thinking skill (the actual ability to perform critical analysis). It is possible that the results would differ if an objective, performance‐based measure of critical thinking ability had been employed. Future research should consider using both disposition and skill measures to capture a more comprehensive picture of nurses' critical thinking competencies. Finally, a key limitation is the modest explanatory power of the regression models, along with the negligible incremental variance when adding overlapping dimensions. This suggests that a considerable portion of the variance remains unexplained; therefore, strong theoretical or practical conclusions may be premature and should be interpreted with caution.

## 9 Implications for Nursing Knowledge and/or Language Development

This study highlights the dimension‐specific nature of the social intelligence–critical thinking relationship, suggesting that nursing frameworks should move beyond reliance on global scores. By integrating psychological‐cognitive terminology, the findings may facilitate clearer communication in education, practice, and interdisciplinary collaborations within competency‐based models.

## Author Contributions

Nahid Sourni performed investigation, data curation, and methodology. Mahvash Kahrizi contributed to validation. Nader Salari conducted formal analysis, methodology and validation. Maryam Janatolmakan wrote the original draft, prepared the visualisations and handled revisions. Rostam Jalali managed conceptualisation, methodology, supervision and project administration. All authors (Nahid Sourni, Mahvash Kahrizi, Nader Salari, Maryam Janatolmakan and Rostam Jalali) participated in writing – review and editing, critically reviewed the manuscript, provided intellectual input, and approved the final version.

## Funding

This thesis project was financially supported by the Research and Technology Deputy of Kermanshah University of Medical Sciences under grant number 4030098. The funding source had no involvement in the design of the study, data collection, data analysis, preparation of the manuscript, or the decision to publish the results.

## Disclosure

The authors used Google Gemini to assist in improving the language clarity, grammar, and readability of this manuscript. The tool was used solely for editing purposes. All research decisions, data interpretation, and final content are the responsibility of the authors.

## Ethics Statement

This study was approved by the Ethics Committee of Kermanshah University of Medical Sciences (Code: IR.KUMS.REC.1403.026). All procedures were performed in accordance with the ethical standards of the 1964 Helsinki Declaration and its later amendments. Written informed consent was obtained from all participants.

## Consent

Written informed consent was obtained from all participants prior to data collection, and participation was entirely voluntary. Confidentiality of data was rigorously maintained throughout the study, with no personal identifiers collected; participants were explicitly assured that their responses would remain anonymous and would not affect their employment status or professional evaluation.

## Conflicts of Interest

The authors declare no conflicts of interest.

## Supporting information


**Data S1:** The STROBE reporting checklist.

## Data Availability

The datasets generated and analysed during the current study are available from the corresponding author upon reasonable request. The questionnaires used in this study were based on validated instruments and are described in detail in the methodology section.
