# Supplementary material for: The Predictive Role of Social Intelligence Dimensions on Critical Thinking Dispositions Among Nurses: A Cross‐Sectional Study in Western Iran
Source: Nurs Open. 2026 Jul 24;13(7):e70676. doi: 10.1002/nop2.70676 (PMC13400976; doi:10.1002/nop2.70676)
Supplement: Supplementary file 1 — Data S1: The STROBE reporting checklist. [file NOP2-13-e70676-s001.docx]

The STROBE reporting checklist

For checking that observational epidemiology research articles can be understood and used by everyone

| How to use this reporting checklist |
| --- |
| This reporting checklist allows authors to demonstrate that their manuscripts adhere to the [STROBE reporting guideline](https:/resources.equator-network.org/reporting-guidelines/strobe/index.html).  If you have not used a reporting guideline before, read about [how and why to use them](https:/resources.equator-network.org/about/reporting-guidelines.html) and check whether STROBE is the [most applicable reporting guideline](https:/resources.equator-network.org/reporting-guidelines/strobe/index.html?#applicability) for your work.  Reporting guidelines are most useful when used early in research. When writing a manuscript or application, consider using the [full guidance](https:/resources.equator-network.org/reporting-guidelines/strobe/index.html) where you’ll find explanations and examples for each item.  After writing, demonstrate adherence by completing this checklist:   1. Specify where each item is described (see [Note 1](#sec-specify)). 2. Cite this checklist (See [Note 2](#sec-cite)). 3. Include your completed checklist as a supplement when submitting to a journal so that future readers can use it to find information. |

|  | Item Description | Location (or reason for not reporting) |
| --- | --- | --- |
| Title and abstract |  | Title: The Predictive Role of Social Intelligence Dimensions on Critical Thinking Dispositions Among Nurses: A Cross-Sectional Study in Western Iran.  **Page 1, lines 1–2** |
| [1a. Indicate the study’s design](https:/resources.equator-network.org/reporting-guidelines/strobe/items/title-abstract-indicate-study-design.html?utm_source=strobe&utm_medium=checklist&utm_campaign=1_1) | Indicate the study’s design with a commonly used term in the title or the abstract. | **Design:** A cross‑sectional descriptive correlational design.  **Page 2, line 32** |
| [1b. Abstract](https:/resources.equator-network.org/reporting-guidelines/strobe/items/abstract.html?utm_source=strobe&utm_medium=checklist&utm_campaign=1_1) | Provide in the abstract an informative and balanced summary of what was done and what was found. | **Page 2, lines 28-57** |
| Introduction |  |  |
| [2. Background / rationale](https:/resources.equator-network.org/reporting-guidelines/strobe/items/background-rationale.html?utm_source=strobe&utm_medium=checklist&utm_campaign=1_1) | Explain the scientific background and rationale for the investigation being reported. | 1 \| Introduction  **Page 4, lines 66-97** |
| [3. Objectives](https:/resources.equator-network.org/reporting-guidelines/strobe/items/objectives.html?utm_source=strobe&utm_medium=checklist&utm_campaign=1_1) | State specific objectives, including any prespecified hypotheses. | 3 \| The Study Objectives  **Page 4, lines 104-109** |
| Methods |  |  |
| [4. Study design](https:/resources.equator-network.org/reporting-guidelines/strobe/items/study-design.html?utm_source=strobe&utm_medium=checklist&utm_campaign=1_1) | Present key elements of study design early in the paper. | 4 \| Method  **Page 5, line 110** |
| [5. Setting](https:/resources.equator-network.org/reporting-guidelines/strobe/items/setting.html?utm_source=strobe&utm_medium=checklist&utm_campaign=1_1) | Describe the setting, locations, and relevant dates, including periods of recruitment, exposure, follow-up, and data collection. | 4 \| Method  4.1 \| Study Design and Settings  4.2 \| Participants and Sampling  4.4 \| Data Collection  Pages 5-7, lines 110-170 |
| [6a. Eligibility criteria](https:/resources.equator-network.org/reporting-guidelines/strobe/items/eligibility-criteria.html?utm_source=strobe&utm_medium=checklist&utm_campaign=1_1) | Cohort study: Give the eligibility criteria, and the sources and methods of selection of participants. Describe methods of follow-up. Case-control study: Give the eligibility criteria, and the sources and methods of case ascertainment and control selection. Give the rationale for the choice of cases and controls. Cross-sectional study: Give the eligibility criteria, and the sources and methods of selection of participants. | **Eligibility Criteria and Sampling Method**  Page 5, lines 118-121 |
| [6b. Matching criteria](https:/resources.equator-network.org/reporting-guidelines/strobe/items/matching-criteria.html?utm_source=strobe&utm_medium=checklist&utm_campaign=1_1) | Cohort study: For matched studies, give matching criteria and number of exposed and unexposed. Case-control study: For matched studies, give matching criteria and the number of controls per case. | **Not applicable. This is a cross‑sectional study; no matching criteria were used.** |
| [7. Variables](https:/resources.equator-network.org/reporting-guidelines/strobe/items/variables.html?utm_source=strobe&utm_medium=checklist&utm_campaign=1_1) | Clearly define all outcomes, exposures, predictors, potential confounders, and effect modifiers. Give diagnostic criteria, if applicable. | All outcomes (critical thinking total and subscales; social intelligence total and subscales) and predictors (social intelligence dimensions, critical thinking dimensions, and demographic/professional characteristics) are clearly defined in the instruments and demographic sections. Diagnostic criteria are not applicable as this is not a clinical diagnostic study; however, categorization boundaries for low/medium/high critical thinking are reported in the results. No formal effect modifiers were tested.  Pages 6-7, lines 136-164 |
| [8. Data sources / measurement](https:/resources.equator-network.org/reporting-guidelines/strobe/items/data-sources-measurement.html?utm_source=strobe&utm_medium=checklist&utm_campaign=1_1) | For each variable of interest give sources of data and details of methods of assessment (measurement). Describe comparability of assessment methods if there is more than one group. | Sections 4.3 (Instruments) and 4.4 (Data Collection) – Methods, all variables are clearly defined with their sources and assessment methods. |
| [9. Bias](https:/resources.equator-network.org/reporting-guidelines/strobe/items/bias.html?utm_source=strobe&utm_medium=checklist&utm_campaign=1_1) | Describe any efforts to address potential sources of bias. | Location: Sections 4.4, 4.5, and 4.6. Description: Bias was addressed through: (a) private and anonymous self‑administration of questionnaires; (b) voluntary participation without incentives; and (c) rigorous testing of statistical assumptions (normality, homogeneity, multicollinearity) before analysis. Self‑report bias is also acknowledged in the limitations. |
| [10. Study size](https:/resources.equator-network.org/reporting-guidelines/strobe/items/study-size.html?utm_source=strobe&utm_medium=checklist&utm_campaign=1_1) | Explain how the study size was arrived at. | 4.2 \| Participants and Sampling  Pages 5-6, lines 117-135 |
| [11. Quantitative variables](https:/resources.equator-network.org/reporting-guidelines/strobe/items/quantitative-variables.html?utm_source=strobe&utm_medium=checklist&utm_campaign=1_1) | Explain how quantitative variables were handled in the analyses. If applicable, describe which groupings were chosen, and why. | **Sections 4.6 (Data Analysis) and 5.2 (Results – for descriptive grouping only).**  All quantitative variables, including age, work experience, average monthly working hours, and total/subscale scores of critical thinking and social intelligence were treated as continuous variables in the primary analyses (Pearson correlations and linear regression models). No grouping was applied to these variables for inferential statistical tests. The only exception is the descriptive categorization of total critical thinking scores into low (33–66), medium (66–99), and high (99–165) levels, which was used solely to present the distribution of participants' competency levels (Section 5.2) and was not used as a predictor in any regression model. |
| [12a. Statistical methods](https:/resources.equator-network.org/reporting-guidelines/strobe/items/statistical-methods-description.html?utm_source=strobe&utm_medium=checklist&utm_campaign=1_1) | Describe all statistical methods, including those used to control for confounding. | 4.6 \| Data Analysis  Page 8, lines 181-193 |
| [12b. Statistical methods – subgroups and interactions](https:/resources.equator-network.org/reporting-guidelines/strobe/items/statistical-methods-subgroups-interactions.html?utm_source=strobe&utm_medium=checklist&utm_campaign=1_1) | Describe any methods used to examine subgroups and interactions. | 4.6 \| Data Analysis  Page 8, lines 181-193 |
| [12c. Statistical methods – missing data](https:/resources.equator-network.org/reporting-guidelines/strobe/items/statistical-methods-missing-data.html?utm_source=strobe&utm_medium=checklist&utm_campaign=1_1) | Explain how missing data were addressed. | No missing data were present for the key study variables. All 442 participants completed the questionnaires in full, and no case was excluded due to incomplete responses. |
| [12di. Statistical methods – loss to follow-up](https:/resources.equator-network.org/reporting-guidelines/strobe/items/statistical-methods-loss-to-follow-up.html?utm_source=strobe&utm_medium=checklist&utm_campaign=1_1) | Cohort study: If applicable, describe how loss to follow-up was addressed. | **Not applicable.** |
| [12dii. Statistical methods – matching cases and controls](https:/resources.equator-network.org/reporting-guidelines/strobe/items/statistical-methods-matching-cases-controls.html?utm_source=strobe&utm_medium=checklist&utm_campaign=1_1) | Case-control study: If applicable, explain how matching of cases and controls was addressed. | **Not applicable.** |
| [12diii. Statistical methods – sampling strategy](https:/resources.equator-network.org/reporting-guidelines/strobe/items/statistical-methods-analytical-methods-sampling-strategy.html?utm_source=strobe&utm_medium=checklist&utm_campaign=1_1) | Cross-sectional study: If applicable, describe analytical methods taking account of sampling strategy. | **Section 4.2 (Sampling) and Section 4.6 (Data Analysis)**  Although stratified random sampling was used to ensure proportional representation across hospitals, the analytical methods did not incorporate sampling weights or adjust for stratification in the regression models. The data were analyzed as a simple random sample, which is consistent with the study's primary aim of examining predictive relationships rather than estimating population parameters. |
| [12e. Statistical methods – sensitivity analyses](https:/resources.equator-network.org/reporting-guidelines/strobe/items/statistical-methods-sensitivity-analyses.html?utm_source=strobe&utm_medium=checklist&utm_campaign=1_1) | Describe any sensitivity analyses. | No sensitivity analyses (e.g., testing robustness to outliers, alternative model specifications, or multiple imputation) were conducted. This is recognized as a limitation of the study. |
| Results |  |  |
| [13a. Participant numbers](https:/resources.equator-network.org/reporting-guidelines/strobe/items/participants-numbers.html?utm_source=strobe&utm_medium=checklist&utm_campaign=1_1) | Report the numbers of individuals at each stage of the study—e.g., numbers potentially eligible, examined for eligibility, confirmed eligible, included in the study, completing follow-up, and analysed; Consider use of a flow diagram. | The total target population consisted of 2,351 nurses across eight hospitals. The required sample size was calculated as 402, with an additional 10% added for potential non‑response, yielding a final target of 442. All 442 nurses were successfully recruited and included in the analysis. No participants were excluded after enrolment, and no one withdrew from the study, as all eligible nurses who were approached agreed to participate and fully completed the questionnaires. Therefore, the number of participants analyzed for each variable remained 442 (or 441 for models involving total social intelligence, as reflected in Table 4).  A flow diagram was not used, as there was no attrition or exclusion after sampling. |
| [13b. Participants – non-participation](https:/resources.equator-network.org/reporting-guidelines/strobe/items/participants-non-participation.html?utm_source=strobe&utm_medium=checklist&utm_campaign=1_1) | Give reasons for non-participation at each stage. | All 442 selected nurses agreed to participate and completed the questionnaires. No refusals or dropouts occurred during data collection. The only predefined reasons for non-participation (mental health disorder or unwillingness) were exclusion criteria applied *before* sampling, but no numbers were excluded because the sampling successfully reached the target size. |
| [13c. Participants – flow diagram](https:/resources.equator-network.org/reporting-guidelines/strobe/items/participants-flow-diagram.html?utm_source=strobe&utm_medium=checklist&utm_campaign=1_1) | Consider use of a flow diagram. | A flow diagram was not used because there was no attrition, loss to follow-up, or post-enrolment exclusion. All 442 recruited participants were directly included in the final analysis, making a flow diagram unnecessary. |
| [14a. Descriptive data – participant characteristics](https:/resources.equator-network.org/reporting-guidelines/strobe/items/descriptive-data-participant-characteristics.html?utm_source=strobe&utm_medium=checklist&utm_campaign=1_1) | Give characteristics of study participants (e.g., demographic, clinical, social) and information on exposures and potential confounders. Present the information in a table. | Participant characteristics (age, gender, marital status, education, work experience, employment status, shift pattern, job rank, department, and monthly working hours) are fully presented in Table 1. Information on exposures (social intelligence and critical thinking dimensions) and potential confounders (demographic/professional variables) is also provided in Section 5.1 and Table 2. |
| [14b. Descriptive data – missing data](https:/resources.equator-network.org/reporting-guidelines/strobe/items/descriptive-data-missing-data.html?utm_source=strobe&utm_medium=checklist&utm_campaign=1_1) | Indicate the number of participants with missing data for each variable of interest. | No missing data were present for any of the primary descriptive variables (demographic characteristics, critical thinking subscales, and social intelligence subscales). All descriptive analyses are based on the full sample of N = 442. (The slight reduction to N = 441 in Table 4 pertains only to the regression model for total social intelligence, as specified in the table footnote; this does not affect the descriptive data.) |
| [14c. Descriptive data – follow-up time](https:/resources.equator-network.org/reporting-guidelines/strobe/items/descriptive-data-follow-up-time.html?utm_source=strobe&utm_medium=checklist&utm_campaign=1_1) | Cohort study: Summarise follow-up time—e.g., average and total amount. | **Not applicable.**  This is a cross‑sectional study; therefore, no follow‑up period was involved. |
| [15. Outcome data](https:/resources.equator-network.org/reporting-guidelines/strobe/items/outcome-data.html?utm_source=strobe&utm_medium=checklist&utm_campaign=1_1) | Cohort study: Report numbers of outcome events or summary measures over time. Case-control study: Report numbers in each exposure category, or summary measures of exposure. Cross-sectional study: Report numbers of outcome events or summary measures. | Summary measures (mean, standard deviation, and range) for total critical thinking and social intelligence scores, along with their subscales, are presented in Table 3. Additionally, the distribution of participants across low, medium, and high critical thinking levels is reported as frequencies and percentages in Section 5.2. |
| [16a. Main results](https:/resources.equator-network.org/reporting-guidelines/strobe/items/main-results.html?utm_source=strobe&utm_medium=checklist&utm_campaign=1_1) | Give unadjusted estimates and, if applicable, confounder-adjusted estimates and their precision (e.g., 95% confidence intervals). Make clear which confounders were adjusted for and why they were included. | **Section 5.4 (Tables 4 and 5)**  Unadjusted estimates (B, β, p‑values) are reported. No confounder‑adjusted estimates or 95% CIs are presented, as the regression models did not include demographic confounders (no covariates were statistically significant in bivariate analyses except job rank, which was not entered as a confounder). |
| [16b. Main results – category boundaries](https:/resources.equator-network.org/reporting-guidelines/strobe/items/main-results-category-boundaries.html?utm_source=strobe&utm_medium=checklist&utm_campaign=1_1) | Report category boundaries when continuous variables were categorised. | Section 5.2 – CT levels: low (33–66), medium (66–99), high (99–165). |
| [16c. Main results – risk](https:/resources.equator-network.org/reporting-guidelines/strobe/items/main-results-risk.html?utm_source=strobe&utm_medium=checklist&utm_campaign=1_1) | If relevant, consider translating estimates of relative risk into absolute risk for a meaningful time period. | Not applicable – cross‑sectional, no risk estimates. |
| [17. Other analyses](https:/resources.equator-network.org/reporting-guidelines/strobe/items/other-analyses.html?utm_source=strobe&utm_medium=checklist&utm_campaign=1_1) | Report other analyses done—e.g., analyses of subgroups and interactions, and sensitivity analyses. | Section 5.1 (subgroup comparisons, Table 2) and Section 5.4 (hierarchical regression, Table 5). No interactions or sensitivity analyses. |
| Discussion |  |  |
| [18. Key results](https:/resources.equator-network.org/reporting-guidelines/strobe/items/key-results.html?utm_source=strobe&utm_medium=checklist&utm_campaign=1_1) | Summarise key results with reference to study objectives. | **Location:** Section 4-1 |
| [19. Limitations](https:/resources.equator-network.org/reporting-guidelines/strobe/items/limitations.html?utm_source=strobe&utm_medium=checklist&utm_campaign=1_1) | Discuss limitations of the study, taking into account sources of potential bias or imprecision. Discuss both direction and magnitude of any potential bias. | 8 \| Limitation  Pages 8-9, lines 415-437 |
| [20. Interpretation](https:/resources.equator-network.org/reporting-guidelines/strobe/items/interpretation.html?utm_source=strobe&utm_medium=checklist&utm_campaign=1_1) | Give a cautious overall interpretation considering objectives, limitations, multiplicity of analyses, results from similar studies, and other relevant evidence. | 7 \| Conclusion  Pages 20-21, lines 403-414 |
| [21. Generalisability](https:/resources.equator-network.org/reporting-guidelines/strobe/items/generalisability.html?utm_source=strobe&utm_medium=checklist&utm_campaign=1_1) | Discuss the generalisability (external validity) of the study results. | Generalizability is discussed in the limitations: the sample was drawn from public university hospitals in a single province (Kermanshah), using convenience sampling. Therefore, findings may not generalize to nurses in private hospitals, non‑academic healthcare settings, or other regions of Iran with different organizational cultures. This is explicitly stated as a limitation, and future multi‑center studies are recommended. |
| Other information |  |  |
| [22. Funding](https:/resources.equator-network.org/reporting-guidelines/strobe/items/funding.html?utm_source=strobe&utm_medium=checklist&utm_campaign=1_1) | Give the source of funding and the role of the funders for the present study and, if applicable, for the original study on which the present article is based. | Pages 23, lines 467-471 |
